# Targeting the Notch1 oncogene by miR-139-5p inhibits glioma metastasis and epithelial-mesenchymal transition (EMT)

**DOI:** 10.1186/s12883-018-1139-8

**Published:** 2018-08-31

**Authors:** Jianlong Li, Qingbin Li, Lin Lin, Rui Wang, Lingchao Chen, Wenzhong Du, Chuanlu Jiang, Ruiyan Li

**Affiliations:** 1 Department of Neurosurgery, The Second Affiliated Hospital of Harbin Medical University, 246 Xuefu Road, Nangang, 150086 Harbin, People’s Republic of China; 2 Department of Orthopaedic Surgery, Nanfang Hospital, Southern Medical University, Guangzhou, 510515 China; 3 Department of Neurology, The Second Affiliated Hospital of Harbin Medical University, Harbin, 150086 China; 4 Neuroscience Institute, Heilongjiang Academy of Medical Sciences, Harbin, 150086 China; 5 Chinese Glioma Cooperative Group (CGCG), Beijing, 100050 China; 6 Department of Neurosurgery, Huashan Hospital, Fudan University, Shanghai, 200040 China; 7 Department of Neurosurgery, The First Affiliated Hospital of Harbin Medical University, Harbin, 150086 China

**Keywords:** miR-139-5p, Notch1, EMT, Glioma

## Abstract

**Background:**

Glioma metastasis, invasion, epithelial-mesenchymal transition (EMT) and chemoresistance indicate poor prognosis. Accumulating evidence reveals that Notch1 is an important factor in tumour progression. However, the role of Notch1 in glioma EMT and associated microRNAs (miRNAs) with the Notch pathway remain controversial.

**Methods:**

Utilizing cBioPortal database to examine the gene signature of NOTCH1 (encoding Notch1), CDH2 (encoding N-cadherin) and SNAI1 (encoding Snail-1) in disease-free survival (DFS) and overall survival (OS). We analyzed the Notch1 expression from Oncomine. We used Western blot (WB), immunohistochemistry (IHC) and immunofluorescence to determine protein levels. Transcription was evaluated by quantitative real-time (qRT)-PCR. siRNA and lentivirus were used to knock down Notch1 and overexpress miR-139-5p, respectively. The migration and invasion of glioma cells were assessed by wound healing and transwell assays. Luciferase reporter assays were utilized to verify the relationship between Notch1 and miR-139-5p. A U87-implanted intracranial model was used to study the effect of miR-139-5p on tumour growth and Notch1 suppression efficacy or EMT reversion.

**Results:**

It revealed the association of NOTCH1, CDH2, SNAI1 genomic alterations with decreases in DFS and OS. Notch1 was upregulated in classical and proneural subtypes of GBM, and associated with tumour grade. Notch1 inhibition suppressed the biological behaviours of metastasis, invasion and EMT. Notch1 was identified as a novel direct target of miR-139-5p. MiR-139-5p overexpression partially phenocopied Notch1 siRNA, whereas the forced expression of Notch1 reversed the effects of miR-139-5p on the invasion of glioma. Moreover, intracranial tumourigenicity and EMT behaviours were reduced by the introduction of miR-139-5p and partially mediated by the decreased Notch1 expression.

**Conclusions:**

miR-139-5p was identified as a tumour suppressor by negatively targeting Notch1, and this work suggests a possible molecular mechanism of the miR-139/Notch1/EMT axis for glioma treatment.

**Electronic supplementary material:**

The online version of this article (10.1186/s12883-018-1139-8) contains supplementary material, which is available to authorized users.

## Background

Glioma is the most common primary malignant tumour of the central nervous system in adults [1, 2]. The high metastasis and invasiveness of glioma induce a high incidence of recurrence, which means a worse prognosis [3]. Epithelial-mesenchymal transition (EMT) includes molecular changes, decreased cell-cell junction and adhesion, and increased cell motility. EMT can be determined by the loss of epithelial markers (E-cadherin) along with the upregulation of mesenchymal markers (N-cadherin, Fibronectin and Vimentin) [4].

Accumulating evidence shows that Notch1 plays an important role in tumour progress. The activation of Notch signalling by tenascin-C promotes the growth of human brain tumour-initiating cells [5]. Notch1 activation is a poor prognostic factor in patients with gastric cancer [6]. Recently, β-carotene has been reported to inhibit EMT though Notch pathway [7]. NOTCH signaling is a primary inducer of EMT in a number of epithelial cancers, including cancer of the lung, breast and pancreas [8]. D Maciaczyk et al. recently demonstrate that blocking Notch-pathway member CBF1 inhibits EMT-activator ZEB1 in glioma cells [9]. However, little is known about the Notch1 interaction with EMT in glioma. Also, the molecular mechanisms remain elusive.

MicroRNAs (miRNAs) are non-coding RNA molecules comprising 18~ 22 nucleotides [10]. They regulate the expression of genes by directly targeting the 3′-untranslated regions (3′-UTR) of corresponding messenger RNAs (mRNAs) [11]. miRNAs are involved in a variety of biological behaviours, including suppressing or promoting tumours. Among these, miR-139 inhibits the growth and metastasis of several cancers including myeloid leukaemia [12], laryngeal squamous carcinoma [13] and liver cancer through targeting, for example, c-Fos and CXCR4 [13, 14]. In particular, miR-139-5p suppresses cancer cell migration by targeting ZEB1 and ZEB2 in glioma [15]. Our previous study confirmed that miR-139 was downregulated in clinical gliomas and glioma cell lines, and miR-139 inhibits Mcl-1 expression and potentiates TMZ-induced apoptosis in glioma [16]. Few reports could be assessed until now, however, regarding the regulation of miR-139 on EMT in glioma, especially though Notch1.

In this study, we attempted to investigate the expression and functions of Notch1 in gliomas and its relationship with miR-139-5p. For the first time, we showed that miR-139-5p reverses the Notch1-mediated EMT of glioma. This suggests an alternative for multiple treatments of glioma by regulating the miR-139-5p/Notch1/EMT pathway.

## Methods

### Patients and specimens

Twenty-nine human glioma tissues and four brain tissues were collected from patients who underwent surgical resection between January 2016 and March 2017 at the Second Affiliated Hospital of Harbin Medical University (HMU). Informed consent was obtained from all patients before the application of their tissue samples. This study complied with the regulations of Declaration of Helsinki and was approved by the medical ethics committee of HMU. All samples were graded histologically by clinical pathologists according to WHO guidelines, and they included 5 grade I tumours, 9 grade II tumours, 5 grade III tumours, and 10 grade IV tumours (Table 1). In addition, 4 normal adult brain tissue specimens were collected from patients who underwent severe traumatic brain injury and required surgical intervention (with informed consent).

**Table 1:** Clinicopathologic parameters of 33 samples from HMU

No.	Age range	Pathology (WHO)	No.	Age range	Pathology (WHO)
01	60–69	Brain Tissue	17	20–29	Pleomorphic Xanthoastrocytoma II
02	50–59	Brain Tissue	18	30–39	Diffuse Astrocytoma II
03	70–79	Brain Tissue	19	40–49	Anaplastic Oligoastrocytoma III
04	50–59	Brain Tissue	20	20–29	Anaplastic Astrocytoma III
05	60–69	Astrocytoma I	21	40–49	Anaplastic Oligoastrocytoma III
06	30–39	Astrocytoma I	22	50–59	Anaplastic Astrocytoma III
07	30–39	Astrocytoma I	23	50–59	Anaplastic Astrocytoma III
08	60–69	Astrocytoma I	24	20–29	Glioblastoma IV
09	40–49	Astrocytoma I	25	50–59	Glioblastoma IV
10	70–79	Oligoastrocytoma II	26	40–49	Glioblastoma IV
11	30–39	Oligoastrocytoma II	27	50–59	Glioblastoma IV
12	40–49	Astrocytoma II	28	40–49	Glioblastoma IV
13	40–49	Diffuse Astrocytoma II	29	30–39	Glioblastoma IV
14	30–39	Diffuse Astrocytoma II	30	50–59	Glioblastoma IV
15	60–69	Diffuse Astrocytoma II	31	70–79	Glioblastoma IV
16	40–49	Oligodendroglioma II	32	60–69	Glioblastoma IV
33	40–49	Glioblastoma IV			

Abbreviation: *WHO* World Health Organization, *HMU* Harbin Medical University

### Cell culture

LN229, U87, T98G and U251 glioma cell lines (human) were purchased from the Chinese Academy of Sciences cell bank. Oligodendroglia (Olig) was a gift from Fengmin Zhang, who is a professor of Harbin Medical University. These cells were cultured in a 5% CO_2_, 37 °C incubator in Dulbecco’s Modified Eagle’s Medium (DMEM, Corning, USA) supplemented with 10% foetal bovine serum (FBS, Biological Industries, Israel).

### MicroRNAs, siRNA and plasmid transfection

MiR-139-5p mimic was purified by high-performance liquid chromatography (GenePharma, Shanghai, China). Notch1 siRNA was composed and purchased from Invitrogen (USA) [17], and the sequences are listed in Additional file 1. The plasmid of full-length Notch1 without the corresponding 3′-UTR, pEGFP-N-Notch1 (GeneChem, Shanghai, China), was amplified and cloned into the GV230 (GeneChem, Shanghai, China). The plasmid along with miR-139-5p mimics or scramble were transfected into glioma cells with Lipofectamine 2000 (Invitrogen, USA) according to the manufacturer’s instructions.

Transfected cells were incubated for another 24–72 h at 37 °C with 5% CO_2_ atmosphere. Afterwards, cells were harvested for RNA and protein analysis.

### In silico analysis and establishing of a three-gene genomic signature

To investigate the potential miRNAs that may regulate Notch1 mRNA, we utilized four commonly used miRNA databases, including miRanda algorithm (http://34.236.212.39/microrna/home.do), miRwalk (http://zmf.umm.uni-heidelberg.de/apps/zmf/mirwalk2/), Pictar (http://www.pictar.org/), and TargetScan (http://www.targetscan.org/vert_71/).

The TCGA data set within the cBioPortal database [18, 19] (http://www.cbioportal.org/index.do) was extracted. The Glioblastoma Multiforme (GBM) cohort (TCGA, Provisional, *n* = 577), a merged cohort (TCGA, Cell 2016, *n* = 1084) of Brain Lower Grade Glioma (LGG) and GBM, the LGG cohort (TCGA, Provisional, *n* = 513) were utilized. NOTCH1 (encoding Notch1), CDH2 (encoding N-cadherin) and SNAI1 (encoding Snail-1) three-gene signature was then examined on independent cohorts above for effects on disease-free survival (DFS) and overall survival (OS).

Notch1 mRNA expression from Oncomine (https://www.oncomine.org/resource/login.html##) and the prognostic meaning of miR-139-5p in glioma from OncoLnc (http://www.oncolnc.org/) was extracted.

### RNA isolation and quantitative real-time (qRT-PCR) assays

Total RNA was picked up using Trizol Reagent (Invitrogen, USA) according to the manufacturer’s instructions. Total cDNA was reversely transcribed from 1 μg of total RNA (Perfect Real Time, Takara, Japan). Two-step qRT-PCR was performed for quantifying gene expression. We used a FastStart Universal SYBR Green Master (ROX) in the Roche LightCycler^R^ Real-Time System. The expression levels were normalized to glyceraldehyde-3-phosphate dehydrogenase (GAPDH) or U6. The PCR conditions started at 95 °C for 15 s, then annealed and extended at 60 °C for 60 s. It is going on for 40 cycles followed by a melting curve analysis. The data was analysed by 2^-ΔΔCt^ method. All experiments were performed in triplicate. The primers used are shown in Additional file 2.

### Western blotting assay

Cell lysates were harvested. Total protein of equivalent amounts were separated by 10% SDS polyacrylamide gel electrophoresis (SDS-PAGE). After that, they were transferred to polyvinylidene difluoride (PVDF) membranes. Block the membranes with 5% fat-free milk and 0.1% Tween-20 in tris-buffered saline with Tween (TBST) for 1.5 h. Next, the membranes were incubated with diluted anti-Notch1 (Abcam), E-cadherin (CST), N-cadherin (CST), Vimentin (Abcam), Fibronectin (Abcam), Snail-1 (Wanleibio), Shh (CST) and anti-GAPDH (Wanleibio) primary antibodies. Anti-rabbit or anti-mouse secondary antibodies (ZSGB-BIO), which were horseradish peroxidase-conjugated, were used and detected by the ECL system (Fujifilm Las-4000).

### Luciferase reporter assay

GV272-Notch1–3′-UTR (Genechem), a wild-type luciferase reporter plasmid was created. It contain a putative miR-139-5p binding sites as previously reported [20]. Using Lipofectamine 2000 reagent (Invitrogen) to transfect these constructs into U87 or LN229 cells, with or without miR-139-5p mimics according to the manufacturer’s protocol. miRNA mimics and firefly luciferase plasmid were co-transfected into cells. For normalization, they were co-transfected with CV045 *Renilla* luciferase plasmid (Genechem, Shanghai, China). Forty hours later, we used Dual-Glo luciferase assay system (E2920, Promega, USA) to measure the luciferase activity. The ratio of Firefly Luciferase activity to that of *Renilla* was defined as normalized luciferase activity.

### Wound healing assay and transwell assay

Cells were plated in 6-well plates. miR-139-5p or Notch1 siRNA were transfected into cells when confluency. A 200-μl sterile pipette tip was used to create scratches. Wash cells twice with PBS and then supply them with DMEM without FBS. Capture photographs at 0 h and after 24–36 h using an Axiovert 200 microscope (Carl Zeiss) and the data was analysed using Image pro-plus software.

Transwell membranes was coated with Matrigel (BD Biosciences, San Jose, CA). About 5 × 10^4^ cells/well were plated in the upper chamber. These cells were treated with miRNAs or Notch1 siRNA. The medium in upper chamber was serum-free. The medium in the lower chamber was 10% FBS. After 24 h, the cells in the top well was removed. The bottom cells were fixed with 95% ethanol, stained with 0.1% crystal violet. Take photographes in three independent 10× fields for each well. Three independent experiments were repeated.

### Immunohistochemistry and immunofluorescence assay

Immunohistochemistry (IHC) and immunofluorescence assays were performed as previously described [21]. IHC scores were assessed using a semiquantitative grading system [22]. The appropriate antibodies against Notch1 (Abcam), E-cadherin (CST), N-cadherin (CST), Fibronectin (Abcam) and Vimentin (Abcam) were used. Immunofluorescence assays were visualized using Goat anti-Rabbit Alexa Fluor® 594-conjugated (ZSGB-BIO) or Goat anti-Mouse Fluorescein–conjugated (ZSGB-BIO) antibodies. Cell nuclei were counterstained using Hoechst 33258 (Thermo Fisher Scientific). Representative images were captured, and they were analysed by Olympus FV1000 Digital laser scanning microscopy.

### Xenograft assay

U87 cells that were co-transducted with miR-139-5p lentivirus/scramble and luciferase lentivirus were injected intracranially into 5-week–old BALB/c-nude mice (Beijing Vital River Laboratory Animal Technology Co., Ltd.) as described earlier [23, 24]. Methods of animal care-taking and feeding were carried out according to the instructions of Beijing Vital River Laboratory Animal Technology (http://www.vitalriver.com/welfare.aspx). Exactly, they were cultured in SPF-class barrier system feeding conditions. The feed was disinfected with 121 degrees, 15 min, 1 kg pressure sterilization. Drinking water was filtered by multiple layers. Each group had 5 mice. After 20 days, the mice were sacrificed exposing to carbon dioxide. Continue to input carbon dioxide at a concentration of 100% for 2 min until the mouse stops breathing and then turns off the switch on the carbon dioxide bottle. Tumours were measured by fluorescent images of whole mice using an IVIS Lumina Imaging System (Xenogen). Portions of the tumour tissues were used to measure the Notch1 and EMT markers by IHC. Cryosections (4 mm) were used for IHC [1, 22]. These procedures were performed with approval by the Harbin Medical University Institutional Animal Care and Use Committee.

### Statistical analysis

SPSS version 13.0 software (Chicago, IL, USA) was used to carried out all statistical analyses. Data were exhibited as means ± SD. Differences between the means of the treatment and control groups were analyzed using student’s t-test. Significance among three or more groups was analyzed by a one-way analysis of variance (ANOVA). Categorical variables were compared using the χ^2^-test and Fisher’s exact test. Data at *p* < 0.05 level were considered statistically significant. The survival curves were analysed using the log-rank test employing GraphPad Prism software.

## Results

### The three-gene signature correlated with decreases in DFS and OS in glioma

**Fig. 1:**
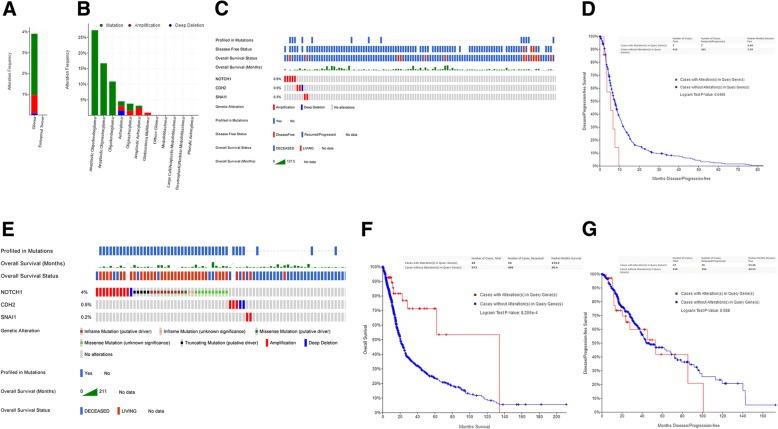
The three-gene signature correlated with decreases in DFS and OS in glioma. (**a**) The comparison of alteration frequency in NOTCH1 between glioma and embryonal tumor. (**b**) The alteration frequency of NOTCH1 in detailed cancer types. (**c**) The indicated types of genomic alterations for the three genes in the TCGA data set (GBM, *n* = 577) within the cBioPortal database are shown; only the proportion of cohorts containing the three-gene signature are included. Each column is for individual tumor. (**d**) Analysis of DFS using the TCGA cohort (GBM) (*P* < 0.05). (**e**) The indicated types of genomic alterations for the three genes in a merged cohort (TCGA, Cell 2016, *n* = 1084) of Brain Lower Grade Glioma (LGG) and GBM within the cBioPortal database are shown; only the proportion of cohorts containing the three-gene signature are included. Each column is for individual tumor. (**f**) Analysis of OS using the TCGA cohort (merged LGG and GBM) (*P* < 0.001). (**g**) Analysis of DFS using the TCGA cohort (*n* = 513, LGG) (*p* = 0.588)

We first explored the alteration frequency of NOTCH1 in different type of brain tumors (*n* = 1300) (cBioPortal) [18, 19] and found the major type of genomic alterations in glioma was mutation or amplication (Fig. 1a and b). Then, we examined NOTCH1, CDH2 and SNAI1 three-gene signature in the Glioblastoma Multiforme (GBM) cohort (TCGA, Provisional, *n* = 577) (Fig. 1c) and demonstrated its association with decreases in DFS (*P* < 0.05) (Fig. 1d). This association was also revealed in a merged cohort (TCGA, Cell 2016, *n* = 1084) of Brain Lower Grade Glioma (LGG) and GBM (*P* < 0.001) (Fig. 1e and f). However, the association was not significant in the LGG cohort (TCGA, Provisional, *n* = 513) (*p* = 0.588) (Fig. 1g).

### Notch1 was upregulated in glioma tissues and cell lines and associated with tumour grade

**Fig. 2:**
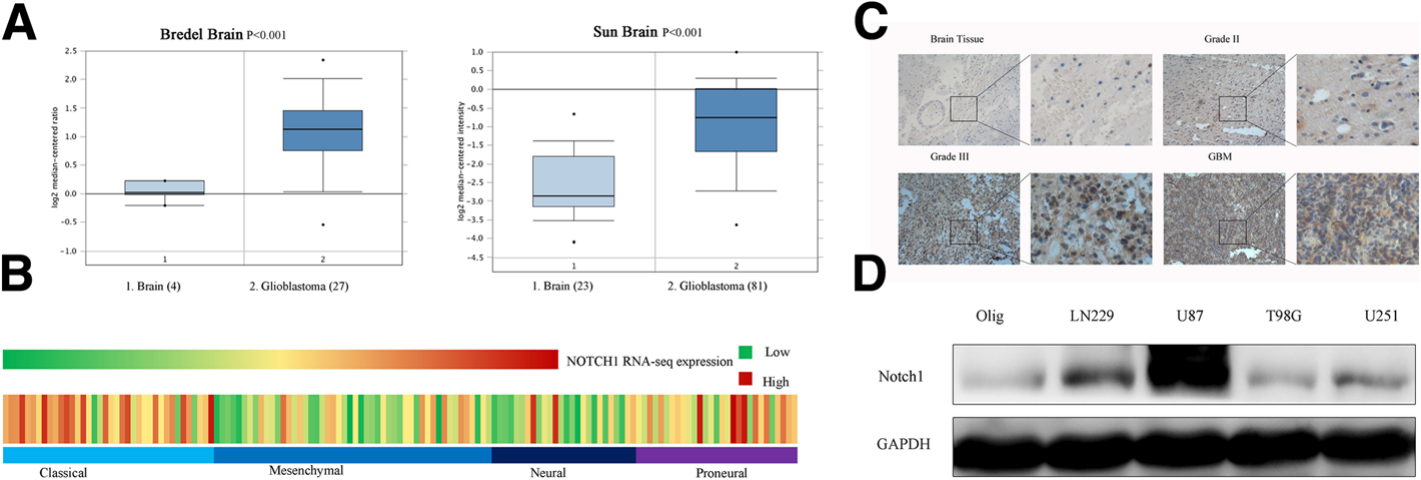
Notch1 was upregulated in glioma and cell lines and associated with tumour grade. (**a**) Notch1 expression was analyzed in GBM tissues and non-tumor brain tissues from the Bredel Brain and Sun brain data sets. (**b**) Notch1 mRNA expression was analyzed in GBM tissues from the TCGA data sets. (**c**) Representative images of Notch1 expression in different grades of glioma tissues and normal brain tissues were shown using immunohistochemical assay (× 100 magnification). (**d**) Western blotting assay showed that T98G, U251, LN229 and U87 glioma cells expressed higher levels of Notch1 than the Olig cell line

We first extracted the data from Oncomine. From Bredel Brain and Sun Brain data sets, we analyzed Notch1 mRNA expression. The result indicated that GBM samples overexpressed more Notch1 (Fig. 2a). Further, we analyzed the mRNA microarray data from TCGA. It suggested that Notch1 were significantly upregulated in classical and proneural subtypes of GBM (Fig. 2b). Notch1 expression was measured using an immunohistochemical analysis in 29 different grades of glioma tissues and 4 normal brain tissues. In Grade III-IV tissues, Notch1 was higher than that in low grade gliomas (WHO II) or normal brain tissues (*P* < 0.05) (Fig. 2c). Furthermore, the expression patterns of Notch1 were confirmed by Western blotting assay in 4 glioma cell lines (LN229, U87, T98G and U251). Glioma cells, especially U87 and LN229 cells, expressed more Notch1 compared with Olig (Fig. 2d). In addition, we assessed the correlation between Notch1 expression and clinicopathologic characteristics in 33 patients and found that Notch1 expression was positively correlated with tumour grade and negatively correlated with Karnofsky Performance status (KPS) score (*P* < 0.05, Table 2).

**Table 2:** Notch1 expression and clinicopathologic characteristics of 33 cases

Variable	Notch1 low expression	Notch1 high expression	*P* value
Gender			*0.711*
Male	6	5	
Female	12	10	
Age (mean/Y)	*48.7*	*48.3*	*0.391*
< 50	11	7	
≥ 50	6	9	
KPS			0.023*
<80	3	10	
≥ 80	14	6	
Grade			0.002*
WHO <II	13	5	
WHO III-IV	2	13	

Abbreviation: *KPS* Karnofsky Performance Status, *WHO* World Health Organization, χ2-test or the Fisher exact test; *statistically significant (*P* < 0.05)

### Notch1 knockdown suppressed metastasis and invasion capability of glioma cells

**Fig. 3:**
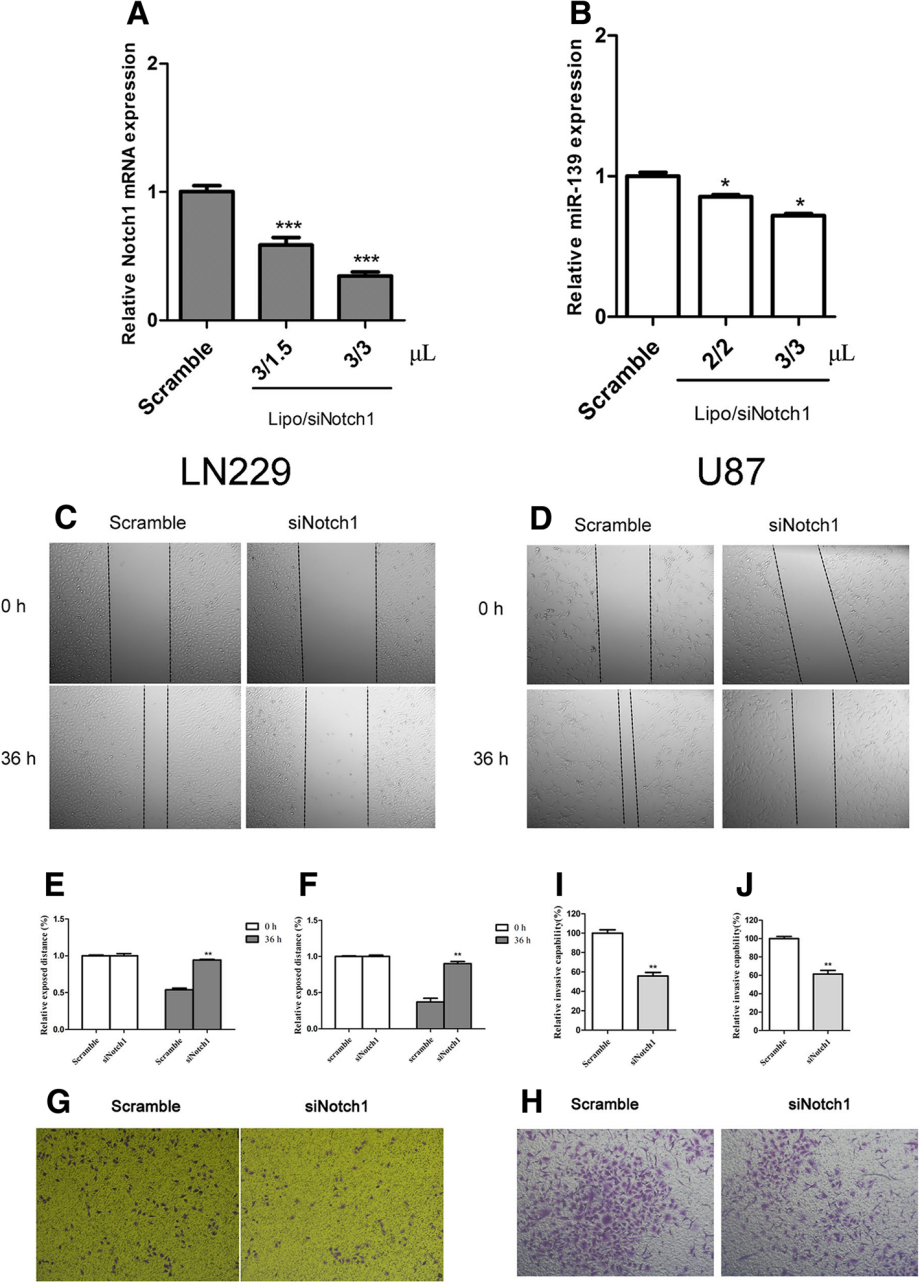
Notch1 knockdown suppressed metastasis and invasion capability of glioma cells. (**a** and **b**) qRT-PCR represented Notch1 expression levels in the LN229 and U87 cells transfected with different quantity of siRNA and lipofectamine2000 (μL. the concentration of siRNA was 20 μM). (**c** and **e**) Wound healing assays confirmed that Notch1 siRNA suppressed the migration of LN229 cells. (× 50 magnification). (**d** and **f**) Wound healing assays confirmed that Notch1 siRNA suppressed the migration of U87 cells. (× 50 magnification). (**g** and **i**) Representative images and histograms of in vitro transwell assays of LN229 after transfected with Notch1 siRNA and control. (× 50 magnification). (**h** and **j**) Representative images and histograms of in vitro transwell assays of U87 after transfected with Notch1 siRNA and control. (× 50 magnification). (*P < 0.05. **P < 0.01. ***P < 0.001)

Given that Notch1 is highly expressed in glioma and a crucial regulator of epithelial-mesenchymal-transition (EMT), we subsequently investigated its biological importance on the tumourigenic property of glioma cells, including metastasis and invasion. We knocked down Notch1 in LN229 and U87 cells (Fig. 3a and b, Fig. 6c and d) and then performed a wound-healing assay and transwell assay to test invasive characteristics. The results showed that siNotch1 attenuated cell migration (Fig. 3c and e for LN229, Fig. 3d and f for U87) and decreased the number of invasive glioma cells compared with the scramble siRNAs (Fig. 3g and i for LN229, Fig. 3h and j for U87).

### Notch1 was a direct target of miR-139-5p

**Fig. 4:**
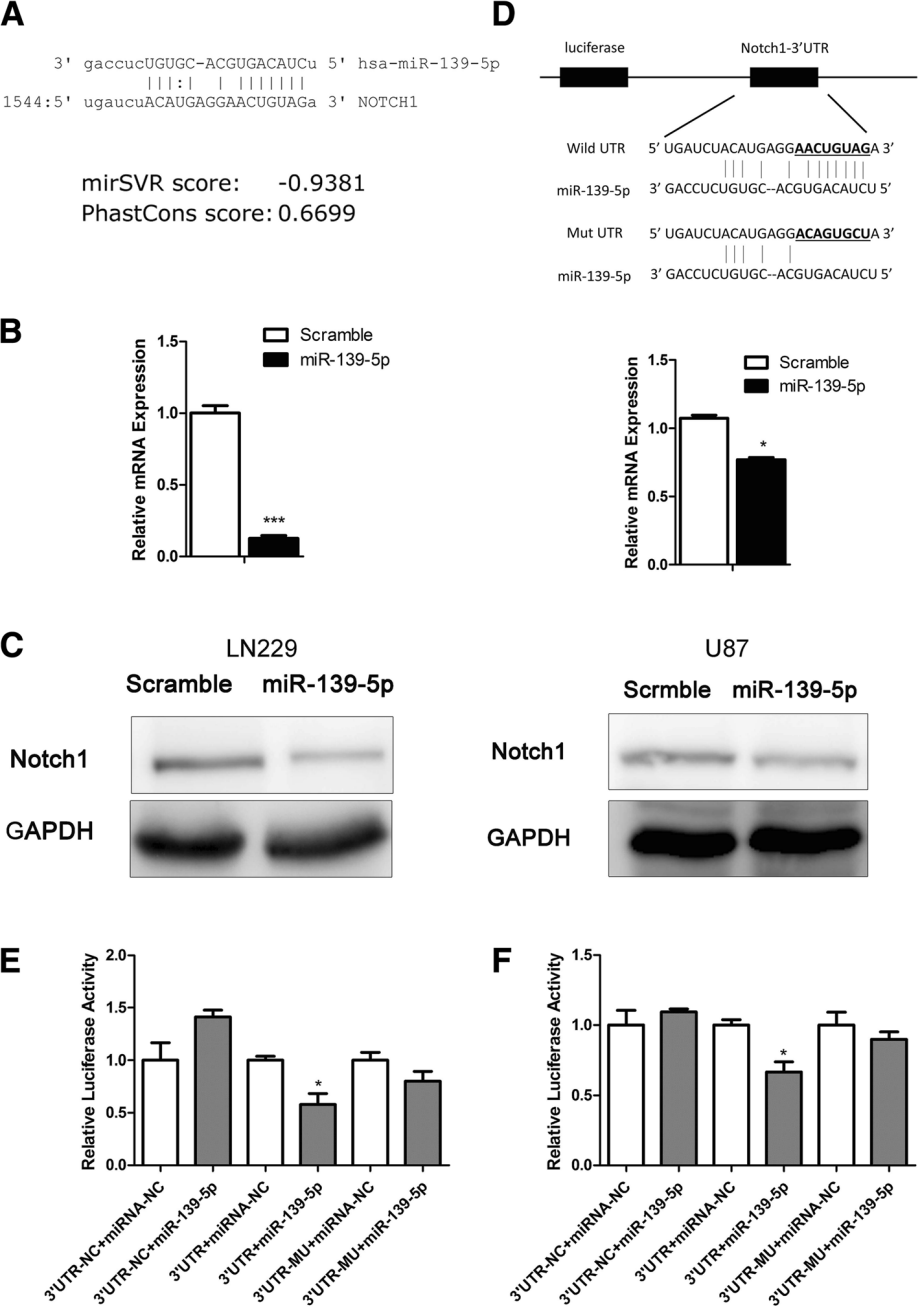
Notch1 was a direct target of miR-139-5p. (**a**) Diagram of the seed sequence of miR-139-5p matched the 3′-UTR of Notch1. (**b** and **c**) qRT-PCR and Western blotting for Notch1 expression after transfection with miR-139-5p or miR-Scr. (*P < 0.05. ***P < 0.001). (**d**) Schematic diagram of the design of wild or mutant Notch1 3′-UTR containing reporter constructs. (**e** and **f**) Luciferase reporter assays in LN229 and U87 glioma cells after co-transfection with wild-type or mutant 3′-UTR Notch1 and miRNAs. 3′-UTR-NC, Negative Control of Notch1 3′-UTR. miRNA-NC, Negative Control of miR-139-5p. The data represent the fold change in the expression (means+SE) of 3 replicates (*P < 0.05)

To investigate whether microRNAs were involved in regulating Notch1, we used the Targetscan, miRanda, Pictar and miRwalk databases and identified potential miRNAs, including miR-139-5p (Fig. 4a), that target Notch1 3′-UTR. Accordingly, we transfected miR-139-5p mimics into glioma cells and evaluated the Notch1 expression level. Transfection efficiency was evaluated using qRT-PCR (Additional file 3). qRT-PCR and Western blotting showed that miR-139-5p induced an obvious decline in Notch1 expression (Fig. 4b and c). Further, we sought to confirm whether Notch1 was a direct target of miR-139-5p. GV272-Notch1–3′-UTR, luciferase reporter plasmid was constructed. It contained a putative miR-139-5p binding site (Fig. 4d). We transfected these plasmids into glioma cells with miRNAs. The data showed that luciferase activity decreased in the group of WT-Notch1–3′-UTR and miR-139-5p mimics. No significant change in any other group (Fig. 4e and f). These data suggest that miR-139-5p binds to the 3′-UTR of Notch1 directly.

### Overexpressed miR-139-5p inhibited glioma metastasis, invasion and EMT

**Fig. 5:**
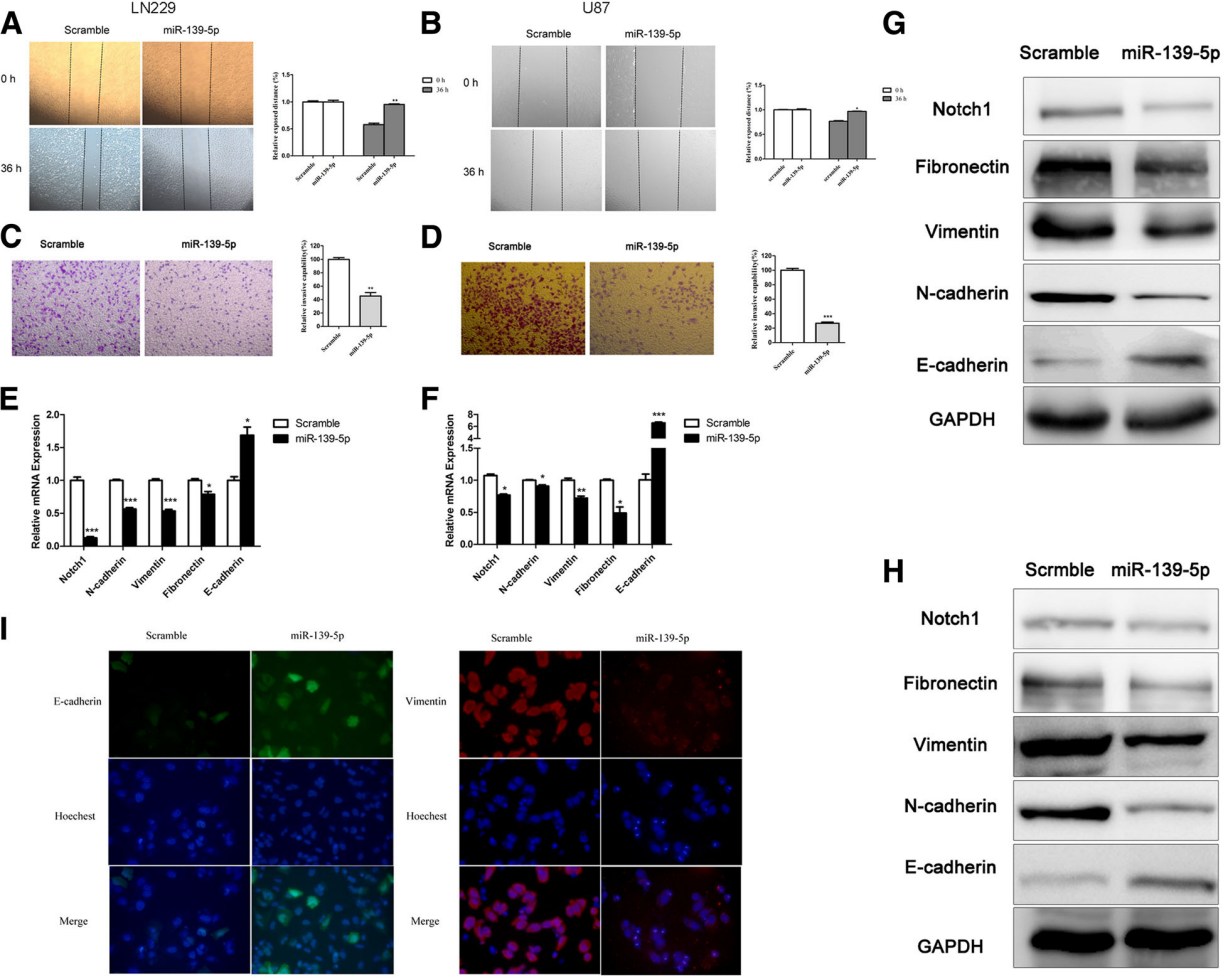
Overexpressed miR-139-5p inhibited glioma metastasis, invasion and EMT. (**a** and **b**) Wound healing assays confirmed that miR-139-5p suppressed the migration of LN229 and U87 cells. (× 50 magnification). (**c** and **d**) Transwell assays indicated that miR-139-5p inhibited LN229 and U87 cell invasion. (× 50 magnification). (**e** and **f**) The mRNA expression levels of different genes. The qRT-PCR analysis demonstrated that the upregulation of miR-139-5p resulted in a reduction of Notch1, N-cadherin, Vimentin and Fibronectin mRNA expression and an elevation of E-cadherin mRNA expression. (**g** and **h**) The Western blot analysis showed that the protein expression of Notch1, N-cadherin, Vimentin and Fibronectin decreased, and the protein expression of E-cadherin increased in LN229 and U87 cells after transfection with miR-139-5p. (**i**) Immunofluorescence showed miR-139-5p overexpression increased E-cadherin expression and decreased Vimentin expression in glioma cells. This experiment was repeated three times. (× 50 magnification). (*P < 0.05. **P < 0.01. ***P < 0.001)

After confirming the relationship between Notch1 and miR-139-5p, we intended to test the effect of miR-139-5p on invasive activity. The wound-healing assay and transwell assay showed that miR-139-5p attenuated cell migration (Fig. 5a and b) and decreased the number of invasive glioma cells compared with the scramble miRNAs (Fig. 5c and d). Moreover, miR-139-5p downregulated the mesenchymal markers (N-cadherin, vimentin and fibronectin) but upregulated the epithelial marker (E-cadherin) at both the mRNA and protein levels (Fig. 5e, f, g, h and i), which indicated an EMT-suppressive role of miR-139-5p.

### Mir-139-5p reversed EMT via down-regulating the expression of Notch1

**Fig. 6:**
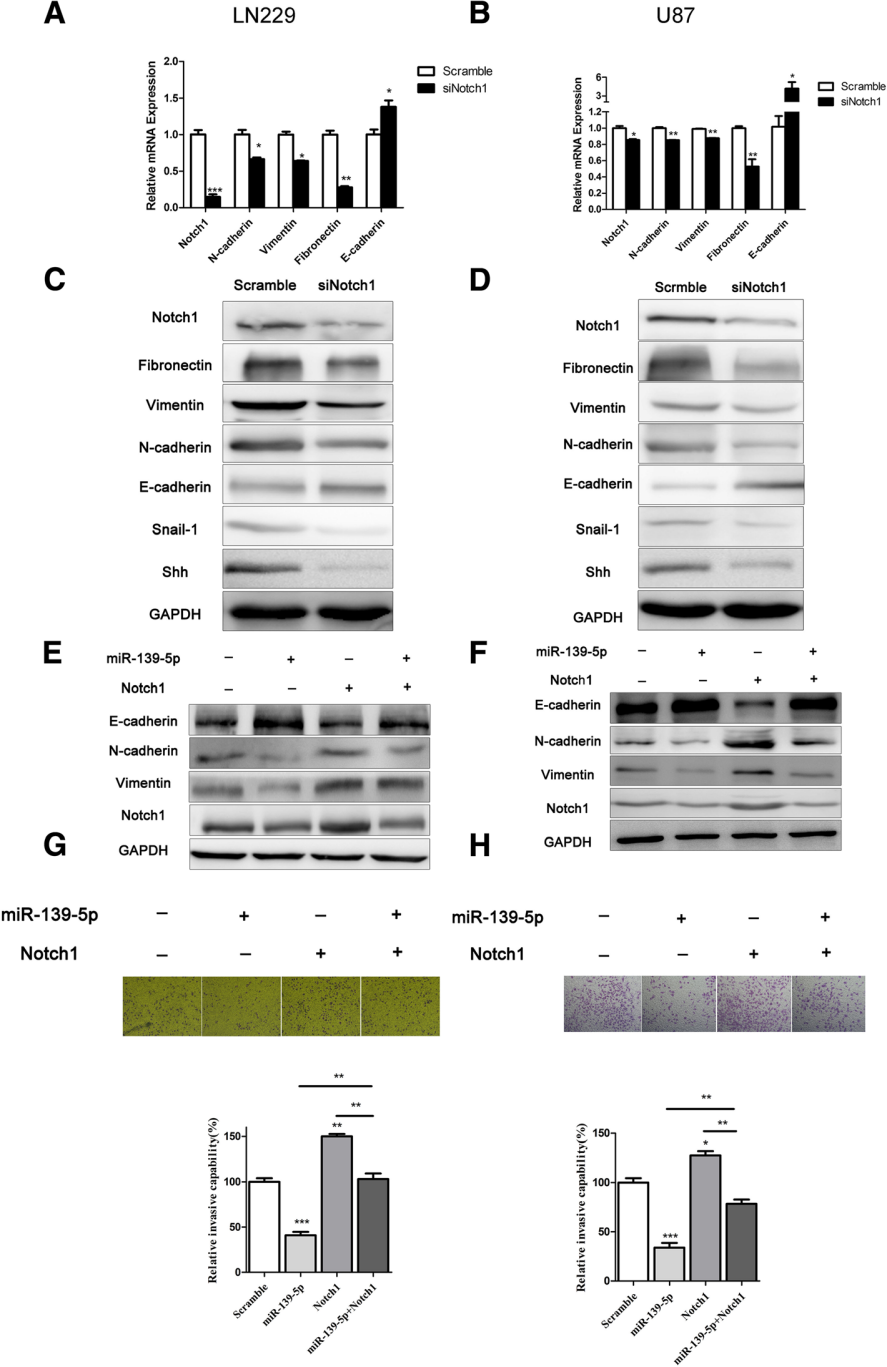
Mir-139-5p reversed EMT via down-regulating the expression of Notch1. (**a** and **b**) qRT-PCR analysis demonstrated that downregulation of Notch1 resulted in a reduction of Notch1, N-cadherin, Vimentin and Fibronectin mRNA expression and an elevation of E-cadherin mRNA expression. (**c** and **d**) Western blot analysis showed that the protein expression of Notch1, N-cadherin, Vimentin, Fibronectin, Snail-1 and Shh decreased, and the protein expression of E-cadherin increased in LN229 and U87 cells treated with Notch1 siRNA. (**e** and **f**) Notch1, E-cadherin, N-cadherin and Vimentin expression levels in the LN229 and U87 cells transfected with full-length Notch1 without the corresponding 3′-UTR or/and miR-139-5p were assessed by Western blotting. (**g** and **h**) Invasiveness of LN229 and U87 glioma cells transfected with full-length Notch1 without the corresponding 3′-UTR or/and miR-139-5p were estimated by transwell assays. This experiment was repeated three times. (× 50 magnification). (*P < 0.05. **P < 0.01. ***P < 0.001)

To further investigate the mechanism of miR-139-5p on glioma suppression, we sought to determine whether the anti-EMT effects of miR-139-5p are mediated by Notch1. To address this, we treated U87 and LN229 with Notch1 siRNA followed by a rescue experiment. The qRT-PCR and Western blotting assay confirmed specific knockdown of Notch1 by siRNA (Fig. 6a-d). Notch1 siRNA dramatically decreased the mesenchymal markers (N-cadherin, vimentin and fibronectin) while increasing the epithelial marker E-cadherin (Fig. 6a-d). Snail-1 is a zinc finger transcription factor that can repress E-cadherin transcription [25, 26]. Sonic hedgehog (Shh) is one of the stem cell-associated protein [27]. The results also showed Notch1 siRNA significantly decreased the expression of Snail-1 and Shh (Fig. 6c and d). In addition, in the treatment with full-length Notch1 without the corresponding 3′-UTR and followed by miR-139-5p mimics for 48 h, we found that miR-139-5p partially inhibits forced Notch1 expression in glioma cells (Fig. 6e and f). Furthermore, the effects of Notch1 on EMT markers after overexpression of miR-139-5p were also examined. The result showed forced expression of Notch1 reversed the effects of miR-139-5p on EMT markers (Fig. 6e and f). Accordingly, the upregulation of Notch1 significantly rescued the glioma invasion behaviour (Fig. 6g and h). Taken together, these data indicated that Notch1 was a mediator of the EMT-suppressive role of miR-139-5p.

### MiR-139-5p inhibited glioma xenograft growth, metastasis and EMT in vivo and prolonged survival

**Fig. 7:**
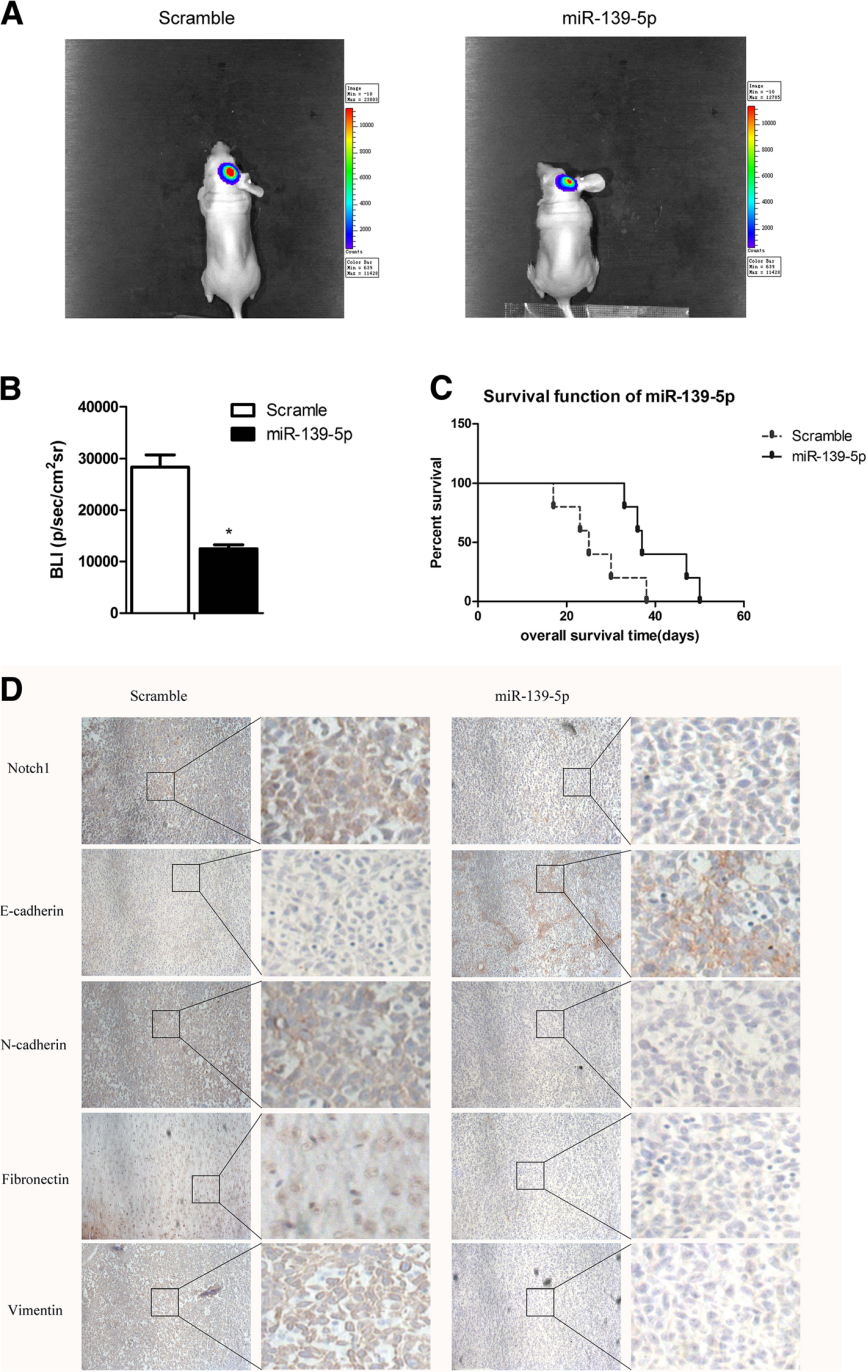
MiR-139-5p inhibited glioma xenograft growth, metastasis and EMT in vivo and prolonged survival. (**a** and **b**) Luminescence imaging for miR-139-5p–treated U87-luc tumours versus scramble-treated controls. (**c**) Kaplan-Meier survival curves indicating that mice transfected with miR-139-5p showed a significantly better outcome than the miR-Scr-treated group (*P < 0.05). (**d**) Notch1, E-cadherin, N-cadherin, Fibronectin and Vimentin expression after transfecting miR-139-5p in tumour sections following IHC analysis. (× 100 magnification)

To evaluate the antiglioma effect of miR-139-5p in vivo, a U87 xenograft model was used. We found that miR-139-5p-treated cells significantly reduced tumour size (*P* < 0.05, Fig. 7a and b). The Kaplan-Meier curve analysis showed a marked longer survival period of the miR-139-5p-treated group compared with the scramble group (*P* < 0.05, Fig. 7c). In the meantime, miR-139-5p decreased the expression of Notch1 as well as mesenchymal markers (N-cadherin, vimentin, fibronectin) while increasing E-cadherin (Fig. 7d).

## Discussion

Glioblastoma is characterized by a high capacity to proliferate and invade. Gliomas that metastasize often have poor prognosis [28, 29]. The search for effective drugs that can suppress glioma metastasis has been a main topic of clinician research. The Notch signalling pathway plays an important role in cell fate determination during normal development [30]. Notch1 has tumour-suppressing and promoting functions in human prostate cancer [31] or in different tumours [5, 32]. A combination of Notch1 blockade and chemotherapy synergistically reduced chemotherapy-enriched cancer stem cells (CSC) [33]. Blocking Notch-1 resulted in downregulation of NF-kappaB and its target genes (*CXCL8*, *MMP9* and *VEGF*), which suppressed invasion and angiogenesis in breast cancer [34]; therefore, we would evaluate these targets next in glioma cell lines and in any other experiments where miR-139-5p levels are manipulated. Sonic hedgehog (Shh) could promote tumour proliferation in a Notch-dependent manner [35–38]. The Hedgehog and Notch pathways interact to control the EMT/MET [35]. However, little is known regarding Notch1 interactions with EMT in glioma. Our study demonstrated that Notch1 was obviously upregulated in glioma tissues. Interestingly, Notch1 also expressed in normal brain tissue, which could be explained by Notch1 signalling being involved in cell fate decision during normal development while abnormal activation would promote carcinogenesis [30]. In addition, Notch1 was upregulated in glioma cell lines, especially U87 and LN229. Knocking down Notch1 in these cells effectively suppressed glioma metastasis, invasion and EMT. These results demonstrated that Notch1 plays an important role in glioma and could be a potential therapeutic target.

MiR-139-5p has been demonstrated as a tumour suppressor in a variety of tumours. Krowiorz et al. found that miR-139-5p is specifically downregulated in CN-AML with mutated FLT3 and acts as a strong tumour suppressor [12]. Wang et al. reported that miR-139 functions as an anti-oncomir to repress glioma progression through targeting IGF-1R, AMY-1, and PGC-1beta [39]. Moreover, miR-139-5p can sensitize colorectal cancer cells to 5-fluororacil by targeting NOTCH-1 [40]. Until now, limited information is available about the effect of miR-139-5p on EMT in glioma. miR-139-5p had prognostic meaning in LGG (Additional file 4). Our recent work demonstrates that miR-139 is downregulated in glioma tissues and negatively correlated to tumour grade [16]. In this study, we searched four databases to find that miR-139-5p may target Notch1 3ˊ-UTR. The combination of bioinformatics prediction, luciferase reporter assays and functional experiments determined that miR-139-5p decreased Notch1 expression and suppressed Notch1-induced EMT in vitro and in vivo. Importantly, miR-139-5p reduced tumourigenicity and prolonged mouse survival. Inhibiting EMT-associated drugs in combination with traditional therapies may provide potential targets for future treatment. There are still important hurdles to overcome such as quick degradation, low efficiency in crossing the blood-brain barrier, side effects and the off-targeting of miR-139-5p.

## Conclusion

Notch1 is markedly overexpressed in glioma and accelerated tumour metastasis, invasion and EMT. Upregulating miR-139-5p in cells inhibits glioma growth and reverses Notch1-induced EMT. This suggests that the miR-139-5p/Notch1/EMT pathway could be a novel target for glioma therapy.

## Additional files

Additional file 1: The oligonucleotide sequences. (PDF 269 kb)

Additional file 2: Gene-specific primers for qRT-PCR analysis. (PDF 261 kb)

Additional file 3: miR-139–5p expression was quantified by qRT–PCR analysis. (PDF 297 kb)

Additional file 4: The clinical prognostic meaning of miR-139-5p in glioma patients with different grade. LGG, brain lower grade glioma. GBM, glioblastoma multiforme. (JPG 632 kb)

## Abbreviations

DFS: Disease-free survival
EMT: Epithelial-to-mesenchymal transition
Hh: Hedgehog
OL: Oligodendroglia cell line
OS: Overall survival
PVDF: Polyvinylidene difluoride
qRT-PCR: Quantitative reverse transcriptase PCR
SDS-PAGE: SDS polyacrylamide gel electrophoresis
Shh: Sonic Hedgehog
