# Supplementary material for: Targeting the Notch1 oncogene by miR-139-5p inhibits glioma metastasis and epithelial-mesenchymal transition (EMT)
Source: BMC Neurol. 2018 Aug 31;18:133. doi: 10.1186/s12883-018-1139-8 (PMC6117922; doi:10.1186/s12883-018-1139-8)
Supplement: Supplementary file 1 — The oligonucleotide sequences. (PDF 269 kb) [file 12883_2018_1139_MOESM1_ESM.pdf]

Table S1. The Oligonucleotides sequences were as follows:

miR-139-5p mimics were designed and purchased from GenePharma (Shanghai, China). miR-139-5p mimics: sense 5´-UCUACAGUGCACGUGUCUCCAGU-3´. anti-sense: 5´-UGGAGACACGUGCACUGUAGAUU-3´. Notch1 siRNA were designed and purchased from Invitrogen (by Life Invitrogen, USA). siRNA sequences were as follows:

Notch-1: 5´ to 3´: r(UGGCGGGAAGUGUGAAGCG)d(TT), r(CGCUUCACACUUCCCGCCA)d(TT). And a scramble siRNA sequence (5´ to 3´: UUCUCCGAACGUGUCACGUTT, ACGUGACACGUUCGGAGAATT) was used as a control.
