# Supplementary material for: Targeting the Notch1 oncogene by miR-139-5p inhibits glioma metastasis and epithelial-mesenchymal transition (EMT)
Source: BMC Neurol. 2018 Aug 31;18:133. doi: 10.1186/s12883-018-1139-8 (PMC6117922; doi:10.1186/s12883-018-1139-8)
Supplement: Supplementary file 2 — Gene-specific primers for qRT-PCR analysis. (PDF 261 kb) [file 12883_2018_1139_MOESM2_ESM.pdf]

Additional file 2. Gene-specific primers for qRT-PCR analysis

**Notch1**	**F 5′- TGAATGGCGGGAAGTGTGAA -3′**	**Fibronectin**	**F 5′- ATCACCCTCACCAACCTCAC -3′**
	**R 5′- ATAGTCTGCCACGCCTCTG -3′**		**R 5′- TCCCTCGGAACATCAGAAAC -3′**
**N-cadherin**	**F 5′- CTGACAATGACCCCACAGC -3′**	**Vimentin**	**F 5′- GAAGGAGGAAATGGCTCGTC -3′**
	**R 5′- TCCTGCTCACCACCACTACTT -3′**		**R 5′- CTCAGGTTCAGGGAGGAAAAG -3′**
**E-cadherin**	**5′- TGATTCTGCTGCTCTTGCTG -3′**	**GAPDH**	**F 5′- TGGACTCCACGACGTACTCAG -3′**
	**R 5′- CTCTTCTCCGCCTCCTTCTT -3′**		**R 5′- CGGGAAGCTTGTCATCAATGGAA -3′**
