# Supplementary material for: Targeting the Notch1 oncogene by miR-139-5p inhibits glioma metastasis and epithelial-mesenchymal transition (EMT)
Source: BMC Neurol. 2018 Aug 31;18:133. doi: 10.1186/s12883-018-1139-8 (PMC6117922; doi:10.1186/s12883-018-1139-8)
Supplement: Supplementary file 3 — miR-139–5p expression was quantified by qRT–PCR analysis. (PDF 297 kb) [file 12883_2018_1139_MOESM3_ESM.pdf]

Additional file 3. miR-139–5p expression was quantified by qRT–PCR analysis.

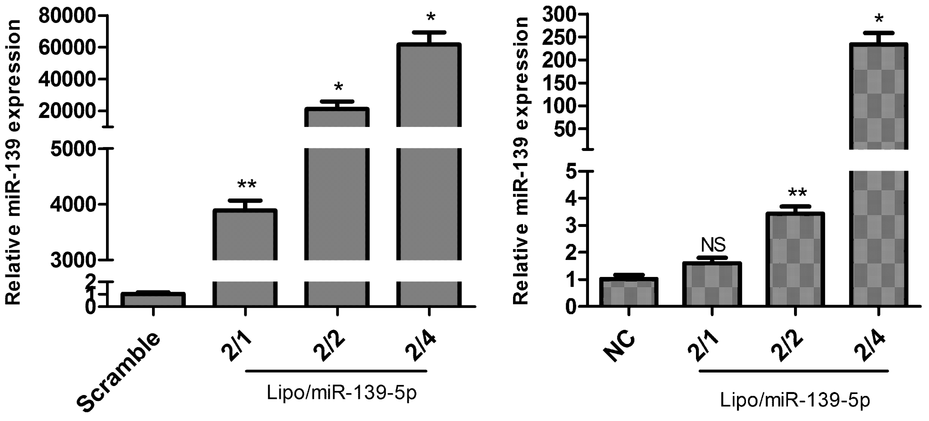

miR-139–5p mimics significantly increased miR-139–5p expression (normalized to U6 RNA) in both LN229 cells (Left) and U87 cells (Right) (*$P < 0.05$. **$P<0.01$. NS, not significant), relative to the control.
