# Supplementary figures and images for: Targeting the Notch1 oncogene by miR-139-5p inhibits glioma metastasis and epithelial-mesenchymal transition (EMT)
Source: BMC Neurol. 2018 Aug 31;18:133. doi: 10.1186/s12883-018-1139-8 (PMC6117922; doi:10.1186/s12883-018-1139-8)

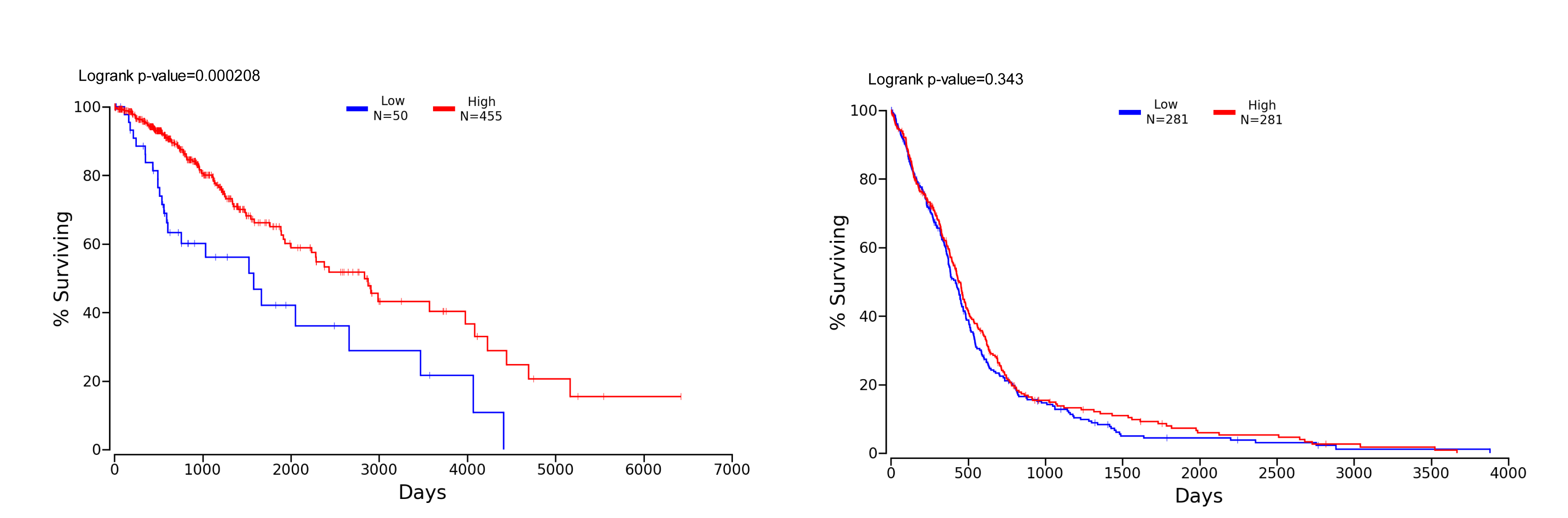

Supplement: Supplementary file 4 — The clinical prognostic meaning of miR-139-5p in glioma patients with different grade. LGG, brain lower grade glioma. GBM, glioblastoma multiforme. (JPG 632 kb) [file 12883_2018_1139_MOESM4_ESM.jpg]
